# Cellulose Nanocrystal and Water-Soluble Cellulose Derivative Based Electromechanical Bending Actuators

**DOI:** 10.3390/ma13102294

**Published:** 2020-05-15

**Authors:** Daniela M. Correia, Erlantz Lizundia, Rafaela M. Meira, Mikel Rincón-Iglesias, Senentxu Lanceros-Méndez

**Affiliations:** 1 Centre of Chemistry, University of Trás-os-Montes e Alto Douro, 5000-801 Vila Real, Portugal; d.correia@fisica.uminho.pt; 2 Centre of Physics, University of Minho, 4710-058 Braga, Portugal; rafaelam_meira@hotmail.com; 3 Department of Graphic Design and Engineering Projects, Bilbao Faculty of Engineering, University of the Basque Country (UPV/EHU), 48013 Bilbao, Spain; 4 BCMaterials, Basque Center for Materials, Applications and Nanostructures, UPV/EHU Science Park, 48940 Leioa, Spain; mikel.rincon@bcmaterials.net (M.R.-I.); senentxu.lanceros@bcmaterials.net (S.L.-M.); 5 IKERBASQUE, Basque Foundation for Science, 48013 Bilbao, Spain

**Keywords:** cellulose nanocrystals, cellulose derivatives, renewable materials, ionic liquid, actuators

## Abstract

This study reports a versatile method for the development of cellulose nanocrystals (CNCs) and water-soluble cellulose derivatives (methyl cellulose (MC), hydroxypropyl cellulose (HPC), and sodium carboxymethyl cellulose (NaCMC)) films comprising the ionic liquid (IL) 2-hydroxy-ethyl-trimethylammonium dihydrogen phosphate ([Ch][DHP]) for actuator fabrication. The influence of the IL content on the morphology and physico–chemical properties of free-standing composite films was evaluated. Independently of the cellulose derivative, the ductility of the films increases upon [Ch][DHP] incorporation to yield elongation at break values of nearly 15%. An increase on the electrical conductivity as a result of the IL incorporation into cellulosic matrices is found. The actuator performance of composites was evaluated, NaCMC/[Ch][DHP] showing the maximum displacement along the x-axis of 9 mm at 8 Vpp. Based on the obtained high electromechanical actuation performance, together with their simple processability and renewable nature, the materials fabricated here represent a step forward in the development of sustainable soft actuators of high practical relevance.

## 1. Introduction

Ionic liquids (ILs) are a diversified group of salts composed of organic cations and a variety of anions that usually present a melting temperature of below 100 °C. This criterion is commonly used to distinguish molten salts and ILs [1]. Their outstanding properties such as low vapor pressure, high thermal, electrochemical and chemical stability, high density, and their inherent ionic conductivity make them suitable for the development of smart and multifunctional materials with new functionalities and applications [2,3]. Particularly, the development of ionic polymer composites based on ILs is receiving increasing attention. When an IL is combined with a polymer, it may give rise to composites that possess electroactive functional properties, leading to IL-based electroactive polymers (EAP) with easy processing, low cost, fracture tolerance, low weight, and mechanical flexibility [4]. IL-based EAPs are finding applications in many different technological fields, in particular for energy harvesting [5], sensors [6], and actuators [7], due to their low operation voltages and a displacement capacity. ILs are good candidates to replace solvents such as water in ionic polymer transducer as their use provides electromechanically active actuators with appropriate actuation voltages and avoid undesired issues related to the loss of the liquid via evaporation [8].

Synthetic polymers such as poly(vinylidene fluoride) (PVDF) and its co-polymers and, a variety of ILs such as, 1-ethyl-3-methylimidazolium bis(trifluoromethylsulfonyl)imide [Emim][TFSI], 1-butyl-3-methylimidazolium hexafluorophosphate [BMIM][PF_6_] [9], (1-hexyl-3-methylimidazolium chloride [C_6_mim][Cl] [4], and 1-ethyl-3-methylimidazolium bis(trifluoromethylsulfonyl)imide [Emim][TFSI], among others, have been used for this type of application. Results demonstrated that the incorporation of the [Emim][TFSI] into the PVDF polymeric matrix induces a plasticizing behavior in the material, strongly influencing its mechanical properties [10]. Moreover, [Emim][TFSI] results in a bending displacement (~3 mm) at low voltages. It has been also shown that the bending actuation response of IL/PVDF composites strongly depend on the proper selection of the IL anion and cation, the actuation depending on anion and cation size [11]. Besides inducing the PVDF crystallization into the highly polar and electroactive β-phase, the incorporation of IL enhances the electrical conductivity and also decreases the Young´s modulus of the polymer. Similar results were obtained by the incorporation of different ILs with a common anion [TFSI]^-^ and distinct cations [12], as well as by the introduction of IL in different polymer matrices such as poly(L-lactic acid) (PLLA) [13], poly(ε-caprolactone) (PCL) [14], and poly(ethylene oxide) (PEO) [15]. 

Despite the obvious interest related to environmental concerns and circular economy regulations, up to now few studies have reported on the development of actuators based on natural polymers. There is nowadays an increased interest in the use of natural polymers as platforms to fabricate multifunctional devices. In this framework, cellulose ((C_6_H_10_O_5_)_n_), the most readily available biopolymer, emerges as an unbeatable candidate. Cellulose-based materials display promising properties to be used in fields as varied as energy storage [16], sensing [17], antimicrobial systems [18], catalytic [19], water purification [20], or tissue engineering [21]. Despite such remarkable efforts, to date scarce works have focused their aim to develop cellulose-based materials with actuator properties, an example being the fabrication of a complex bilayer system consisting on mesoporous photonic resins using cellulose nanocrystals (CNCs) [22]. Additionally, Nevstrueva et al. showed that cellulose can be in soft artificial muscle applications because of its flexibility, low driving voltage, and inherent biocompatibility [23].

As a novel cellulose-derivative, CNCs are nanoscale spindle-shaped crystals extracted upon the controlled hydrolysis of native cellulose [24]. The ability of CNCs to form iridescent chiral nematic structures was used to obtain materials with stimuli-responsive actuation behavior and variation in photonic properties [22]. Similarly, a multi-layered structure using CNCs and polyethylene glycol diacrylate has been developed to obtain actuator properties upon swelling [25]. However, these approaches consist of complex fabrication processes, making their scalability a serious drawback. In order to improve natural polymer-based actuators’ performance and processability, the present work reports on CNC-based actuators using simple fabrication technologies: CNCs were mixed through evaporation induced self-assembly (EISA) with a biocompatible ionic liquid, 2-hydroxy-ethyl-trimethylammonium dihydrogen phosphate ([Ch][DHP]).

As CNCs are known to be brittle and the flexibility of the actuator is a key factor determining its performance, the use of other cellulose-based materials is also evaluated. Importantly, methyl cellulose (MC), hydroxypropyl cellulose (HPC), and sodium carboxymethyl cellulose (NaCMC) have been used, which apart from presenting an elongation at break exceeding 10%, have remarkable film forming ability [26]. Therefore, CNC, MC, HPC, and NaCMC have been blended with IL contents up to 40% wt. *via* solvent casting approach using [Ch][DHP] as a model IL. Film morphology, electrical, mechanical, and electro-mechanical properties are evaluated and the potential of cellulose/[Ch][DHP] films as actuator materials demonstrated.

## 2. Materials and Methods 

### 2.1. Starting Materials

Microcrystalline cellulose with an average particle size of 20 μm (310697-500G), sulphuric acid (95–97%, 30743-1L, Sigma-Aldrich, St. Louis, MO, USA), hydroxypropyl cellulose with a *M_w_* of 100,000 g·mol^−1^ (HPC, 191884-100G, Sigma-Aldrich, St. Louis, MO, USA), methyl cellulose powder with a viscosity of 400 cP when dissolved at 2 wt.% in water at 20 °C (MC, M0262-100G, Sigma-Aldrich, St. Louis, MO, USA) and sodium carboxymethyl cellulose with a *M_w_* of 250,000 g·mol^−1^ and a degree of substitution of 1.2 (NaCMC, 419281- 100G, Sigma-Aldrich, St. Louis, MO, USA) were supplied by Sigma Aldrich. The IL 2-hydroxyethyl-trimethylammonium dihydrogen phosphate ([Ch][DHP]) (purity higher than > 98%) [Ch][DHP] was acquired from Iolitec (Heilbronn, Germany). 

### 2.2. Sample Preparation

Cellulose nanocrystals (CNCs) were extracted from microcrystalline cellulose through sulphuric acid hydrolysis followed by tip-sonication. Firstly, 20 g of microcrystalline cellulose were subjected to hydrolysis with 64% (w/w) sulphuric acid solution (400 mL) at 45 °C for 30 min (400 rpm). The reaction was then quenched by adding 10-fold excess of cold distilled water. The remaining suspension was washed by centrifugation (4000 rpm) to remove excess aqueous acid. CNCs were obtained by sonication in a Vibracell Sonicator (Sonics & Materials Inc., Newton, CT, USA) at 50% output for 10 min. After further purification by dialysis, aqueous CNC dispersions with a pH of 1.9 and a concentration of 1.46% w/w were obtained.

**Figure 1:**
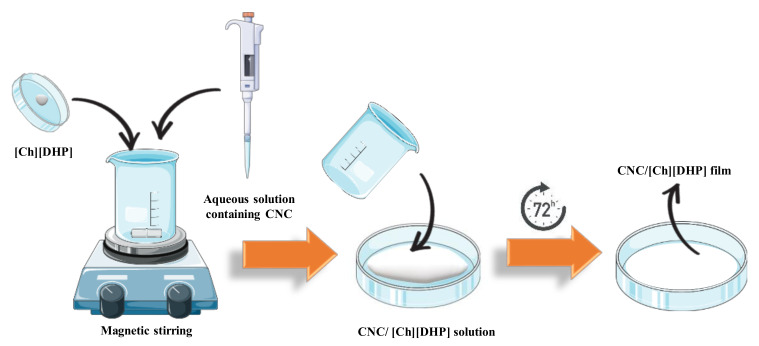
Schematic representation of cellulose-derivatives/[Ch][DHP] composite films prepared by solvent evaporation method.

Nanocomposites based on CNCs and 2-hydroxyethyl-trimethylammonium dihydrogen phosphate [Ch][DHP] were prepared by the solvent evaporation method (Figure 1). Different contents of [Ch][DHP] (0, 10, 25, and 40% wt.) were added to an aqueous solution containing CNC and mixed at room temperature under magnetic stirring. After the complete IL dissolution, the CNC/[Ch][DHP] solutions were cast in Petri-dishes and dried at room temperature for 72 h. Similarly, for the water-soluble cellulose derivatives/[Ch][DHP] films, the IL (40% wt.) was previously dispersed into ultra-pure water, followed by the cellulose derivatives (HPC, MC and NaCMC) dissolution. Similarly, to the neat CNC and CNC/[Ch][DHP], the pure cellulose derivatives and cellulose derivatives/[Ch][DHP] films were obtained after the water evaporation at room temperature. The thickness of the samples is shown in Table S1.

### 2.3. Characterization

The samples were coated with gold by magnetron sputtering (Polaron SC502, Quorum Technologies, Lewes, UK) and their morphology was evaluated by scanning electron microscopy (SEM), in a NanoSEM – Fei Nova 200 Microscope (FEI Company, Hillsboro, OR, USA) with an Everhart–Thornley detector (ETD) at an accelerating voltage of 10 kV.

A Jasco FT/IR-4100 FTIR (JASCO International, Hachioji, Japan) was used for Fourier transform infrared spectroscopy (FTIR) measurements in attenuated total reflection (ATR) mode. Sixty-four scans were recorded at room temperature in the range between 600 and 4000 cm^−1^ with a resolution of 4 cm^−1^. 

X-ray powder diffraction (XRD) patterns were measured using a Bruker D8 Advance diffractometer (Bruker Corporation, Billerica, MA, USA) equipped with a Cu tube, Ge(111) incident beam monochromator (l ¼ 1.5406 Å) (fixed slit 1 mm), and a Sol-X energy dispersive detector (fixed slit 0.06 mm). The samples were mounted on a zero background silicon wafer embedded in a generic sample holder.

The volume electrical conductivity of the films was obtained by measuring the current–voltage (I–V) characteristic curves at room temperature applying voltages from −10 to 10 V using an automated Keithley 487 picoammeter/voltage source (Keithley Instruments, Beaverton, OR, USA). Prior to the measurements, circular gold electrodes of 5 mm diameter were coated in both sides by magnetron sputtering (Scancoat Six SEM Sputter Coater, HHV, Bangalore, India). The electrical conductivity was evaluated from the I–V slope applying the Equation (1) [27]:

(1) σ=1R×AL

where R is the resistance, A is the electrode area, and L is the thickness of the samples. 

Room temperature mechanical measurements were carried out in an AG-IS universal testing machine (Shimadzu, Kyoto, Japan) with a load cell of 50 N at a stretching rate of 1 mm·min^−1^. Rectangular samples (20 × 10 mm) with thicknesses between 15 and 34 µm were used to perform these measurements. 

For the evaluation of the bending response a procedure similar to the one presented by Dias et al. was used [28]. In short, samples of 12 mm × 2 mm were gold coated by magnetron sputtering (Scancoat Six SEM Sputter Coater, HHV, Bangalore, India) onto both sides. The needles of the sample holder were connected to an Agilent 33220A function generator (Keysight Technologies, Santa Rosa, CA, USA) and an oscilloscope PicoScope 4227 (Pico Technology, Cambridgeshire, UK). All the samples were analyzed by a square wave signal with different peak voltages (2, 3, 5, 8, and 10 Vpp) at a frequency of 100 mHz. Used IL is electrochemically stable in the operation window here analyzed [1]. The electromechanical response of the actuators was performed at room temperature under environmental conditions and no effect of ambient humidity was considered [29].

## 3. Results and Discussion

### 3.1. Morphological Characterization

**Figure 2:**
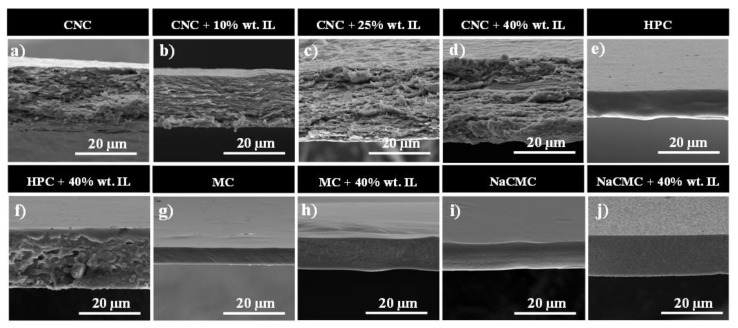
Scanning electron microscope (SEM) images of: (**a**) pure cellulose nanocrystals (CNC); (**b**) CNC + 10% wt. ionic liquid (IL); (**c**) CNC + 25% wt. IL; (**d**) CNC + 40% wt. IL; (**e**) pure HPC; (**f**) hydroxypropyl cellulose (HPC) + 40% wt. IL; (**g**) pure methyl cellulose (MC); (**h**) MC + 40% wt. IL; (**i**) pure sodium carboxymethyl cellulose (NaCMC); and (**j**) NaCMC + 40% wt. IL.

The morphology of the prepared films has been studied by scanning electron microscopy (SEM). Figure 2 shows the cross-section SEM micrographs of neat CNC, HPC, MC, and NaCMC films, together with their IL composite counterparts. Neat CNC (Figure 2a) presents a characteristic parallel arranged layered structure formed upon the evaporation induced self-assembly (EISA) of CNCs, which organize into a long-range chiral nematic order [30,31,32]. Interestingly, although the structure is not completely homogeneous at large IL loadings (Figure 2b–d) its incorporation does not disrupt the layered structure of the CNC film, suggesting a rather homogeneous infiltration of the ionic liquid between individual nanocrystals [33].

The fractured edge of neat HPC, MC, and NaCMC films (Figure 2e, g, and i, respectively) shows homogeneous surfaces with no irregularities, confirming their suitable film-forming properties. Upon IL addition, both MC and NaCMC films still keep a uniform and flat morphology, while HPC/IL films present a non-homogeneous and rough surface. This effect is explained in terms of a poor interaction of the non-ionic HPC with [Ch][DHP], which yields non-uniform films with voids during solvent evaporation. As observed in the micrographs, all the films present a uniform thickness ranging from 15 to 30 μm (see Table S1 for further details).

It should be taken into account that due to the sulphuric acid hydrolysis, CNC surfaces are decorated with anionic sulphate ester groups (-OSO_3_^−^), yielding nanoparticles comprising approximately 0.6% to 0.8% of elemental sulphur by weight (zeta-potential values of nearly −40 mV). Therefore, CNCs are covered by a negative electrostatic charge, rendering acidic pH values when dispersed in water [24]. In order to obtain homogeneous free-standing films with no IL degradation (which in turn may yield decreased functional properties), such IL should be compatible with acidic pH values. To ensure no negative effects when mixing water-dispersed CNCs with IL, [Ch][DHP] was therefore selected, as it is slightly acidic due to the proton-donating ability of the DHP anion [34].

### 3.2. Molecular and Crystalline Structure

**Figure 3:**
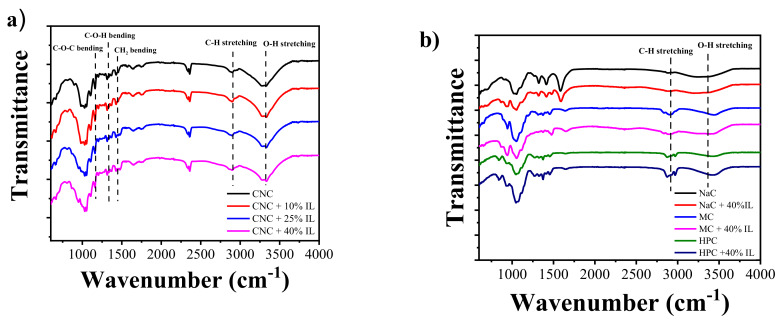
Fourier transform infrared spectroscopy (FTIR)-attenuated total reflection (ATR) spectra of (**a**) pure CNC and CNC/[Ch][DHP] composite films and (**b**) pure cellulose derivative and cellulose derivative/[Ch][DHP] films containing 40% wt. of IL.

Fourier transform infrared (FTIR) spectroscopy has been used to study the presence of chemical interactions between CNC, HPC, MC, or NaCMC and the [Ch][DHP] ionic liquid. As depicted in Figure 3, all samples present a broad band located at 3600–3200 cm^−1^ arising from the O−H stretching and a weaker band at 2902 cm^−1^ due to the C−H stretching, which is a characteristic feature of cellulose derivatives [35]. More precisely, CNC is characterized by further bands at 1430 cm^−1^ (CH_2_ bending), at 1337 cm^−1^ (C-O-H bending), at 1160 cm^−1^ (C-O-C bending), and at 897 cm^−1^ (C-O-C asymmetric stretching) [36]. MC shows bands at 1646 cm^−1^ (C-O stretching), 1455−1251 cm^−1^ (C-H bending), 1065 cm^−1^ (C-O stretching), and 947 cm^−1^ (O-CH_3_ stretching) [37]. Finally, NaCMC has bands at 1605 cm^−1^ (asymmetrical stretching of carboxyl methyl ether), 1422 cm^−1^ (symmetrical stretching of carboxyl methyl ether), and 1128 cm^−1^ (>CH-O-CH_2_) [38]. No band shifting is observed upon IL incorporation, suggesting that no chemical interaction is achieved between the different cellulose types and the [Ch][DHP] ionic liquid.

From the FTIR-ATR spectra of the CNC/[Ch][DHP] composite films the main absorption bands characteristics of the [Ch][DHP] IL are also observed, being an indicative of the IL incorporation into the CNC and its derivatives. The absorption band at 950 cm^−1^ is attributed to the stretching vibration of the -CCO group, increasing the absorption band intensity with increasing IL content (Figure 3a). The [Ch][DHP] characteristic absorption bands at 1479 cm^−1^, ascribed to the CH_3_ vibration, and at 1644 cm^−1^, assigned to the O-H vibration, are identified in the spectra [39]. 

**Figure 4:**
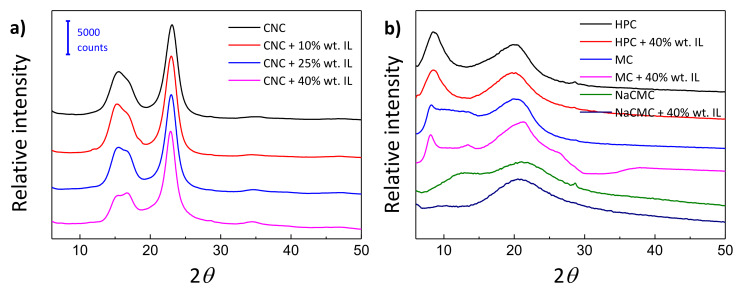
X-ray diffraction (XRD) patterns of (**a**) pure CNC and CNC/[Ch][DHP] composite films and (**b**) HPC, MC, and NaCMC-based composite films.

X-ray diffraction (XRD) analyses were conducted to study the influence of [Ch][DHP] incorporation into the crystalline structure of the resulting films. Figure 4 depicts XRD patterns for CNC/IL films for [Ch][DHP] concentration up to 40% wt. All the films show the characteristic diffraction peaks corresponding to cellulose I, with four main peaks located at 2*θ* = 14.9°, 16.5°, 22.7°, and 34.4° corresponding to *(1–10)*, *(101)*, *(200)* and *(004)* planes [40]. Although [Ch][DHP] could form long-range order chloline crystals with sharp diffraction peaks in the 2*θ* range of 10° to 35° (the most intense ones being located at 2*θ* ≈ 22.5° and 35°) no reflections corresponding to [Ch][DHP] are noticed irrespectively of its concentration, suggesting that the presence of CNC hinders the formation of such chlorine crystals [41].

The XRD patters corresponding to neat and composite water-soluble cellulose derivatives with an IL concentration of 40% wt. are presented in Figure 4. Again, in spite of the presence of large amounts of [Ch][DHP], no diffraction patterns corresponding to chlorine crystals are observed, indicating that [Ch][DHP] remains in its amorphous state in all the prepared films. HPC and MC present two broad peaks at 2*θ* = 8.8° and 20.1° and 2*θ* = 8.2° and 20.3°, respectively, corresponding to *(1–10)* and *(110)* planes, similarly to cellulose II [42]. Interestingly, the crystalline structure of HPC and MC is not disrupted upon [Ch][DHP] incorporation. On the contrary, when [Ch][DHP] is added to NaCMC, its original semi-crystalline character (as evidenced by the two broad diffraction peaks centered at 2*θ* = 13.0° and 21.1°) is suppressed [43], suggesting that the presence of ionic liquids limits the ability of NaCMC to form crystalline regions.

HPC and MC maintain the crystalline characteristic of cellulose II as a result of their low degree of substitution (despite to their slightly larger *d*-spacing of the *(1–10)* plane). On the contrary, NaCMC presents a more amorphous character due to presence of larger carboxymethyl ether group in the sodium salt form (-O-CH_2_-COO^−^Na^+^). It is also observed that all the films composed by cellulose derivatives (with or without [Ch][DHP]) present broad diffraction peaks, indicating the coexistence of both crystalline and amorphous phases within the films.

### 3.3. Mechanical Properties

**Figure 5:**
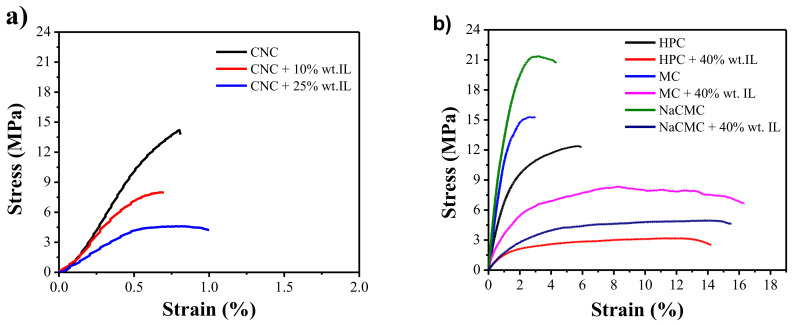
Representative stress-strain curves obtained for (**a**) neat CNC and [Ch][DHP]/CNC composites with different [Ch][DHP] contents (10 and 25% wt.) and for (**b**) neat cellulose derivatives and cellulose derivative/[Ch][DHP] composites containing 40% wt. of IL.

The determination of the mechanical properties of the developed free-standing films is essential for understanding their potential for electromechanical actuator applications. Accordingly, as summarized in Figure 5 and Table 1, room-temperature uniaxial tensile tests have been used to determine the Young’s modulus (*E*), maximum tensile stress (*σ_y_*), and elongation at break (*ε_b_*). As observed in Figure 5a, neat CNC film shows its characteristic brittle and stiff behavior with an *E*, *σ_y_*, and *ε_b_* of 2394 ± 215 MPa [33], 14.27 MPa, and 0.8%, respectively. Upon IL addition, the stiffness of the films decreases showing a noticeable plastic deformation region (in contrast with neat CNC film, which only shows a linear section corresponding to elastic deformation), which is accompanied by a marked decrease in *E* down to 589 ± 290 MPa and in *σ_y_* down to 465 MPa for the CNC + 25% wt IL sample (*ε_b_* remains barely unchanged). This fact is attributed to the plasticizing effect of the IL, which penetrates between adjacent CNCs to reduce their hydrogen bonding-induced entanglement. Despite the observed decrease in *E* when in the presence of IL, it should be noticed that the modulus of the CNC/IL films still remains above the values reported for some commodity thermoplastics such as polyethylene terephthalate (PET) [44].

All three cellulose derivatives studied follow a similar trend than the CNC/IL films (Figure 5b). Neat HPC presents a semi-ductile behavior with an *E* and *ε_b_* of 919 ± 71 MPa and 5.6%, respectively, while MC and NaCMC show a semi-brittle behavior with an *E* and *ε_b_* of 1284 ± 183 and 1889 ± 323 MPa and 2.8% and 4.3%, respectively [45]. IL addition increases the elongation at break and the ductility of the resulting HPC, MC, and NaCMC films (Table 1), inducing a plasticizing effect in the [Ch][DHP], and then a thermoplastic behavior followed by a linear regime [12]. Typically, the IL incorporation limits the hydrogen bonding between cellulosic chains, reducing chain rigidity and increasing the elongation at break of the films, decreasing the Young Modulus. These experimental results show that it is possible to obtain films with tailored mechanical properties, ranging from stiff and brittle to flexible and ductile, by controlling cellulose type (CNC, HPC, MC, or NaCMC) and IL concentration.

### 3.4. Electrical Properties

**Figure 6:**
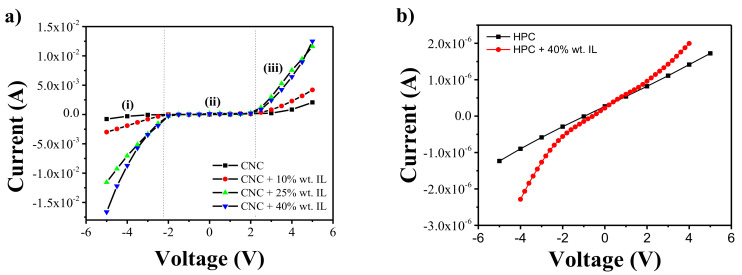

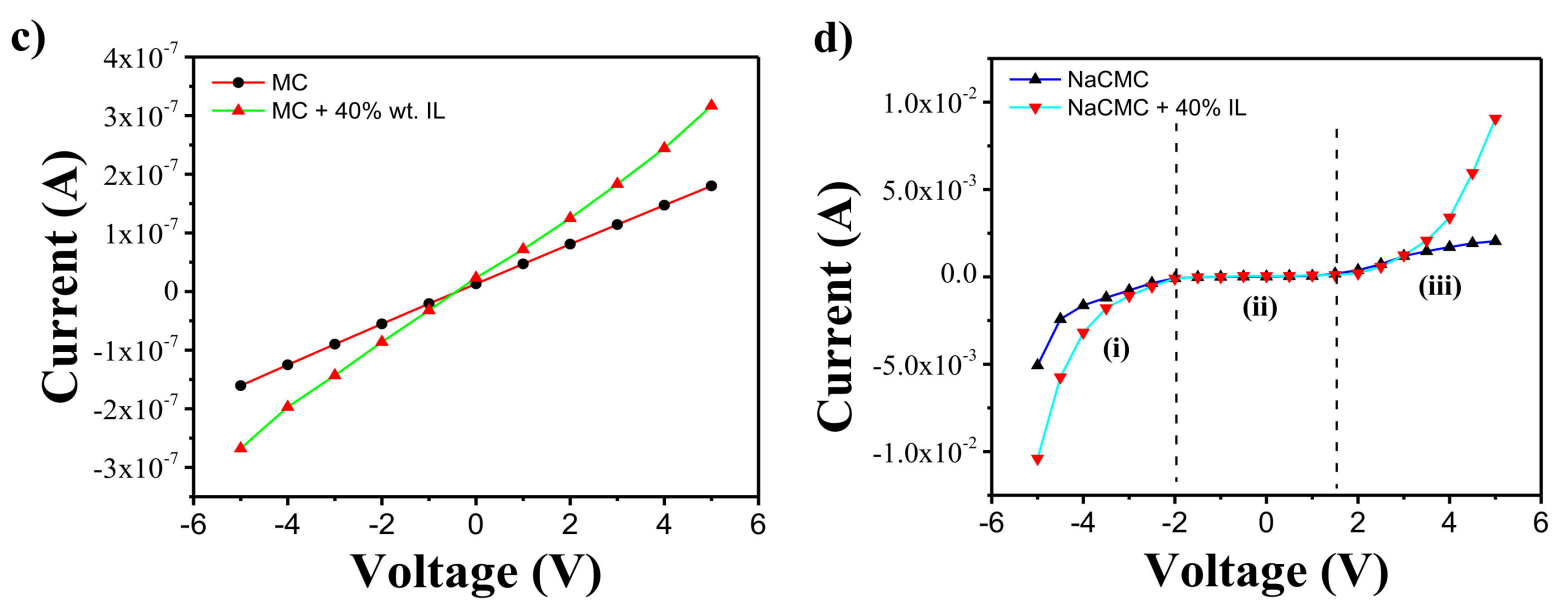
Representative current-voltage (I–V) curves for (**a**) neat CNC and CNC/[Ch][DHP] composite films with different IL contents (10, 25 and 40% wt.), (**b**) neat HPC and HPC + 40% wt. IL, (**c**) neat MC and MC + 40% wt. IL and (**d**) neat NaCMC and NaCMC + 40% wt. IL.

The effect of the IL incorporation in the electrical conductivity of the [CH][DHP]/CNC composites and its derivative s was evaluated from the current-voltage curves presented in Figure 6. Figure 6a shows that neat CNC presents non-linear I–V curves, characterized by two regimes: one between −2 and 2 V and one above those voltages. This behavior is more noticeable for the CNC/[Ch][DHP] samples indicating a hindrance of IL cation and anion movement within the CNC polymer matrix, attributed to stronger electrostatic interactions between IL and the polymer matrix. For an applied voltage higher than 2 V, the electrical field is strong enough to induce the cation and anion movement, yielding a stronger increase of the electrical conductivity. This behavior is also observed for neat NaCMC and NaCMC/[Ch][DHP] composites. For neat HPC and MC and its composites, on the contrary, a linear curve is observed with the applied voltage. 

The effect of the [Ch][DHP] in the electrical conductivity of all developed samples was quantified from the I–V curves of Figure 6 using Equation (1), and the corresponding conductivity values are shown in Table 2. Independently of the cellulose derivative, the incorporation of [Ch][DHP] into the polymer matrix improves the electrical conductivity of the composites, increasing the electrical conductivity with increasing IL content. It is notable that this increase occurs in all three regimes, but it is particularly relevant for regime iii, being therefore determined by the higher content of charged species (anions and cations). On the other hand, a lower increase with filler content is observed for regimes i and iii, the increase of electrical conductivity being attributed to increased mobility of the charge’s species. Interestingly, for the CNC and NaCMC, the increase in the electrical conductivity in the regimes i and iii does not follow a symmetrical behavior, provably due to the polarity of surface interactions. The highest conductivity value is observed for the CNC/[Ch]DHP] samples containing 40% wt. of IL, showing a conductivity value of 6.43 × 10^−3^ ± 3.53 × 10^−4^ S·m^−1^ (regime iii). 

### 3.5. Bending Actuation Performance

**Figure 7:**
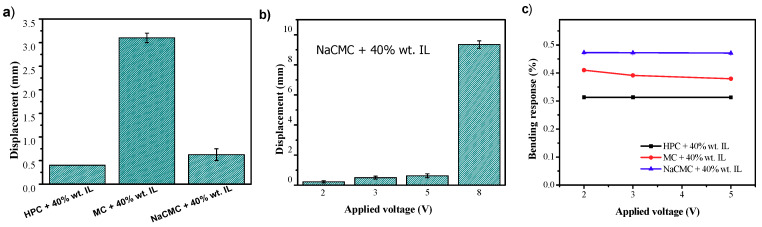
Displacement of the composites for (**a**) an applied voltage of 5 V at a frequency of 100 mHz for HPC, MC, and NaCMC composites containing 40% wt. of IL and (**b**) as a function of the applied voltage for the NaCMC + 40% wt. IL composite at 100 mHz. (**c**) Bending response as a function of the applied voltage for the different cellulose derived composites.

**Figure 8:**
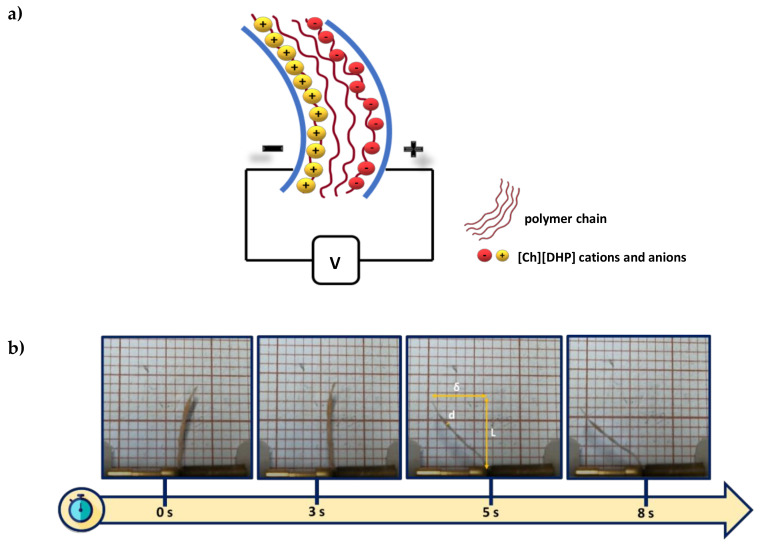
(**a**) Schematic representation of the cation and anion migration and (**b**) bending response as a function of time for NaCMC/[Ch][DHP] composite at a 100 mHz frequency and 8 V.

The electromechanical properties of the CNC/[Ch][DHP] and cellulose derivatives films was evaluated measuring the maximum displacement under the applied electric field, which is measured from the distance of the tip of the actuator from the relaxed position (Figure 7 and Figure 8). For CNC/[Ch][DHP] composites, low displacements are obtained (0.2 mm) (results not shown) due to the mechanical properties of the [Ch][DHP]/CNC composites, when compared with the water-soluble cellulose derivatives. As shown in Figure 5 and Table 2, CNC composites show a brittle and stiff behavior with a higher Young Modulus when compared with the CNC derivatives composites, therefore hindering the ions’ mobility. Figure 7a shows the maximum displacement for the water-soluble cellulose derivative/[Ch][DHP] composites with an applied voltage of 5 V at a frequency of 100 mHz. MC/[Ch][DHP] films developed a maximum displacement of 3.4 mm, larger than the one obtained for the HPC/[Ch][DHP] and NaCMC/[Ch][DHP] composites. On the other hand, this composite does not show bending actuation for applied voltages larger than 5 V, probably due to its lower conductivity values when compared with the HPC and NaCMC/[Ch][DHP] composites. Further, MC/[Ch][DHP] composites shows the highest Young Modulus (5.70 ± 0.98 MPa) hindering the bending motion.

The displacement as a function of the applied voltage is shown for the NaCMC/[Ch][DHP] samples (Figure 7b), showing an increase of the maximum displacement up to 9.3 mm with increasing applied voltage up to 8 V (see video in supplementary information S1). The observed bending response is due to the presence of anions and cations from the [Ch][DHP], their diffusion and displacement upon an applied voltage, to the electrode layers (positive and negative electrode side, respectively), and to the different sizes of anion and cation [11], finally leading to the bending of the film. Thus, bending actuation is determined both by the electrical and the mechanical properties of the films [12], as schematized in Figure 8a. The bending motion as a function of time for the NaCMC/[Ch[DHP] composite for an applied voltage of 8 V at a frequency of 100 mHz is presented in Figure 8b and in supplementary information (Video S1). The largest displacement is observed for the NaCMC/[Ch][DHP] composites, indicating that the NaCMC matrix favors the ion diffusion and mobility as a result of the high ion-dipole interactions between the [Ch][DHP] with the NaCMC polymer chain. The IL-polymer interactions decrease from the NaCMC<MC to the HPC, being indicative of the Na^+^ interactions with the IL anions. Further, as expected, the highest bending responses are observed for these types of composites, followed by the MC and HPC composites due to the lowest Young Modulus (NaCMC<HPC<MC), as demonstrated in Table 1. It is notable that the NaCMC/[Ch][DHP] composites present the highest conductivity values, favoring the movement of IL charge in response to an electric field. Both these parameters favor the bending motion of the films, resulting in higher bending responses.

Finally, the bending response (ε) of the HPC/[Ch][DHP], MC/[Ch][DHP] and NaCMC/[Ch][DHP] composites was evaluated for applied voltages between 2 to 10 V through Equation (2), where “*L*” is the sample free length, “*d*” the thickness, and “*δ*” the displacement along the x-axes (see the schematic representation of Figure 8a for the representation of the main parameters).

(2) $$\epsilon =\frac{2\times d\times \delta }{L^{2}+\delta^{2}}$$

It is noteworthy that the highest bending response is observed for the NaCMC-based films as a result of the highest observed displacements comparatively to the other sample’s displacement response, suggesting that NaCMC presents a bright future to develop electromechanical materials.

## 4. Conclusions

This work describes the development of cellulose-based free-standing films for actuator applications. CNC and three water-soluble cellulose derivative free-standing films comprising the IL [Ch][DHP] were developed by a solvent casting method. Different contents of [Ch][DHP] (10, 25 and 40% wt.) were incorporated within the CNC biopolymer matrix. In the base of the obtained results, the highest IL content (40% wt.) was also incorporated into the HPC, MC and NaCMC matrix. No morphological changes were observed for neat and CNC, MC, and NaCMC composites upon the IL incorporation, all the samples presenting a smooth and flat surface. The mechanical properties evaluated from the uniaxial tensile measurements revealed a plasticizing effect as a result of the IL incorporation into the polymer matrix, resulting in ductile free-standing films. For all the samples the Young Modulus decreases with the IL incorporation into the matrix, and the electrical conductivity of the composites increases together with the IL concentration, showing, in the case of CNC and NaCMC, two electrical conductivity regimes based on the increase mobility of anions and cations for applied voltages larger than +/− 2 V. Bending actuators were developed, the highest bending motion of ~9 mm being obtained for the NaCMC/[Ch][DHP] composite at a frequency of 100 mHz and an applied voltage of 8 V, thus highlighting the suitability of the developed systems for environmentally-friendly high performance actuators. 

## Figures and Tables

**Table 1:** Main mechanical parameters obtained after uniaxial tensile test for the prepared free-standing films.

Sample	*E* (MPa)	*σ_y_* (MPa)	*ε_b_* (%)
CNC	2394 ± 215	14.27	0.8
CNC + 10% wt. IL	1461 ± 74	14.31	0.7
CNC + 25% wt. IL	589 ± 290	4.65	1.0
HPC	919 ± 71	12.38	5.6
MC	1284 ± 183	15.26	2.8
NaCMC	1889 ± 323	21.27	4.3
HPC + 40% wt. IL	249 ± 45	2.52	14.1
MC + 40% wt. IL	570 ± 98	6.59	16.3
NaCMC + 40% wt. IL	208 ± 28	5.07	15.6

*E*: Young’s modulus; *σ_y_*: stress at yield; and *ε_b_*: elongation at break.

**Table 2:** Electrical conductivity of the neat cellulose derivatives and their IL-based composites.

Sample	Electrical Conductivity (S·m^−1^)		
	(i)	(ii)	(iii)
CNC	1.02 × 10^−7^ ± 0.0	3.82 × 10^−9^ ± 0.0	1.40 × 10^−7^ ± 0.0
HPC	-	2.42 × 10^−7^ ± 0.0	-
MC	-	2.22 × 10^−8^ ± 0.0	-
NaCMC	6.52 × 10^−4^ ± 0.0	2.17 × 10^−5^ ± 3.82 × 10^−6^	4.34 × 10^−4^ ± 3.87 × 10^−5^
CNC + 10% wt. IL	9.61 × 10^−4^ ± 1.40 × 10^−4^	4.39 × 10^−5^ ± 3.87 × 10^−6^	1.32 × 10^−3^ ± 6.53 × 10^−5^
CNC + 25% wt. IL	5.50 × 10^−3^ ± 1.18 × 10^−4^	1.29 × 10^−4^ ± 1.41 × 10^−5^	6.09 × 10^−3^ ± 1.22 × 10^−4^
CNC + 40% wt. IL	8.62 × 10^−3^ ± 3.45 × 10^−4^	1.21 × 10^−4^ ± 2.44 × 10^−5^	6.43 × 10^−3^ ± 3.53 × 10^−4^
HPC + 40% wt. IL	-	2.79 × 10^−7^ ± 3.99 × 10^−8^	-
MC + 40% wt. IL	-	5.75 × 10^−8^ ± 1.02 × 10^−8^	-
NaCMC + 40% wt. IL	1.25 × 10^−3^ ± 1.18 × 10^−4^	4.82 × 10^−5^ ± 0.0	2.37 × 10^−3^ ± 3.15 × 10^−4^

## Supplementary Materials

The following are available online at https://www.mdpi.com/1996-1944/13/10/2294/s1, Table S1: Film thickness, Video S1: Bending actuation performance of the NaCMC/[Ch][DHP] composite.
