# Supplementary material for: Cellulose Nanocrystal and Water-Soluble Cellulose Derivative Based Electromechanical Bending Actuators
Source: Materials (Basel). 2020 May 15;13(10):2294. doi: 10.3390/ma13102294 (PMC7287802; doi:10.3390/ma13102294)
Supplement: Supplementary file 1 [file materials-13-02294-s001.zip › materials-799341-s.docx]

Supplementary Materials

**Table S1.** Thickness (µm) of the samples.

| Sample | Thickness (µm) |
| --- | --- |
| CNC | 28.7 ± 3.1 |
| HPC | 15.8 ± 2.4 |
| MC | 14.5 ± 2.1 |
| NaCMC | 16.0 ± 2.6 |
| CNC + 10% wt. IL | 16.2 ± 1.5 |
| CNC + 25% wt. IL | 29.2 ± 1.6 |
| CNC + 40% wt. IL | 33.8 ± 2.5 |
| HPC + 40% wt. IL | 15.7 ± 1.1 |
| MC + 40% wt. IL | 21.2 ± 2.9 |
| NaCMC + 40% wt. IL | 23.7 ± 3.5 |
